# Comparison of contrast-to-noise ratios of different detection methods in ultrasound optical tomography

**DOI:** 10.1364/BOE.457075

**Published:** 2022-08-18

**Authors:** Alexander Bengtsson, David Hill, Kevin Shortiss, Lars Rippe, Stefan Kröll

**Affiliations:** 1 Lund University, Atomic Physics Division, Department of Physics, Professorsgatan 1, Lund, 22363, Sweden; 2 SpectraCure AB, Gasverksgatan 1, Lund, 22229, Sweden

## Abstract

Ultrasound optical tomography (UOT) is a hybrid imaging modality based on interaction between ultrasound and light, with a potential to extend optical imaging capabilities in biological tissues to depths of several centimeters. Several methods have been developed to detect the UOT signal. To better understand their potential for deep tissue imaging, we present a theoretical contrast-to-noise comparison between the spectral hole burning, single-shot off-axis holography, speckle contrast, and photorefractive detection methods for UOT. Our results indicate that spectral hole burning filters have the potential to reach the largest imaging depths. We find that digital off-axis holography and photorefractive detection can have good contrast-to-noise ratio at significant depths. The speckle contrast method has a smaller penetration depth comparatively.

## 1. Introduction

Optical imaging provides molecular contrast, and is thus widely used for biomedical applications. Conventional optical techniques, however, suffer from severe image blurring with increasing imaging depth due to light scattering, which is the predominant light-tissue interaction. Ultrasound optical tomography (UOT) is a technique that aims at performing spatially resolved optical measurements at several centimeter depths in biological tissue [1,2]. Simulations indicate that UOT could image deeper into tissue than photoacoustic tomography [3], which makes the technique interesting to further investigate.

**Fig. 1.**
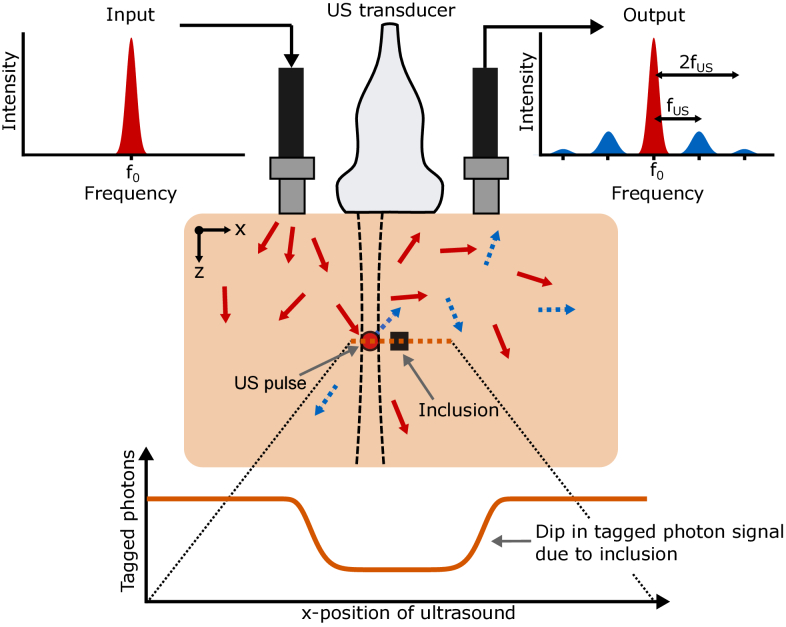
Illustration of the principle of conventional ultrasound optical tomography (UOT) imaging. An ultrasound (US) pulse is propagated into tissue to a depth of interest. The tissue is then illuminated with laser light. The US pulse frequency shifts a small proportion of the photons which propagate through the US pulse volume. This frequency shift is equal to a multiple of the US pulse frequency $f_{\text{US}}$ . These frequency shifted photons (often called tagged photons, represented by blue arrows above) and non-shifted photons (untagged photons, represented by red arrows above) are both collected. By measuring the change in the tagged photons signal one may obtain spatial information about the optical absorption at the ultrasound location deep within tissue.

In UOT, diffuse photons traversing a spatially localized ultrasonic pulse inside tissue are phase modulated (and thereby frequency shifted) due to the periodic refractive index variation and scatterer movement caused by the ultrasonic wave, in a similar way to a light pulse passing through an acousto-optic modulator. These ultrasound-modulated photons are often called tagged photons. Conventional reflection mode UOT imaging is illustrated in Fig. 1. The number of tagged photons at the tissue surface depends on the local photon fluence, and the optical and acoustic properties of the volume where the ultrasound pulse is located. By systematically scanning the ultrasound while recording the tagged photon signal, an image can be reconstructed. Detecting the weak tagged signal over the much stronger background of untagged photons at the original laser frequency is, however, challenging. A plethora of signal detection methods have been developed, but it is still an open task to determine the most suitable methods for different types of future UOT devices and medical applications. Incoherent methods include confocal Fabry-Pérot cavities [4,5] and spectral hole burning filters [6–11]. In these methods, the filter passband is matched with the frequency of the tagged photons. The untagged photons outside the passband are greatly attenuated. Several coherent methods have also been developed to detect tagged photons. Early experiments used a fast single-point detector to measure the intensity modulation of speckles at the ultrasound frequency [12,13]. However, each speckle oscillates with a random phase. Increasing the detector area and thereby the number of speckles impinging on a single-point detector does therefore not improve the signal-to-noise ratio (SNR), since both the integrated heterodyne signal and noise scale as the square root of the number of detected speckles [12]. This leads to poor SNR. Efforts have therefore been directed towards methods where multiple speckles are processed in parallel using multi-element detectors to improve the SNR. Li et al. and others used the ultrasound induced change in laser speckle contrast [14–19]. Different holography and interferometry techniques employing a reference beam with a frequency matching either the tagged or untagged photons have been used [20–23]. There are also holographic methods that do not require multi-pixel detectors, such as photorefractive detection [24–31], and recently a method based on laser feedback interferometry has also shown promising results [32].

The coherent methods are sensitive to speckle decorrelation, i.e., loss of temporal coherence of speckles due to internal tissue movement, e.g. blood flow. The speckle decorrelation time in human tissues is ∼ 0.1-1 ms [33]. Moreover, the decorrelation time decreases with imaging depth, as shown for in vivo mouse brain tissue [34]. Thus, coherent UOT methods targeting several cm imaging depths have to be fast, and the acousto-optic signal has to be acquired within the speckle decorrelation time to not suffer SNR degradation.

In this paper, a theoretical study of the contrast-to-noise ratio (CNR) of some selected UOT methods is presented. We consider: (a) spectral hole burning filters [6–11], as the method is insensitive to speckle decorrelation and has large detection etendue (this method is what the authors of this paper are presently investigating experimentally, see also the disclosure paragraph after the conclusion); (b) photorefractive detection due to the impressive imaging depths presented in the literature using phantoms with tissue-like optical properties [28]; (c) single-shot off-axis holography [20] to represent digital holographic methods, with the particular version considered here requiring only a single camera exposure to acquire an acousto-optic signal, thus minimizing SNR degradation due to speckle decorrelation; and (d) speckle contrast methods [14–19] because they have been frequently used, and have the advantage of a comparatively simple setup.

We find that spectral hole burning filters have the potential to achieve the best CNR scaling. When combined with the large detection etendue of the hole burning method, this should translate into the largest imaging depths of the investigated methods. We also find that off-axis holography and photorefractive crystals can have good CNR at significant depths. According to our calculations, the speckle contrast method performs poorly compared to the other methods, which is attributed to the random nature of speckles.

## 2. Calculating contrast-to-noise

To compare various detection methods, we are interested in estimating how well they can distinguish different tissues, e.g. a tumor or ischemia from the background tissue. The CNR can be used for this purpose, and is in this paper defined as:

(1) $$\text{CNR}=\frac{\text{Contrast}}{\text{Noise}}=\frac{|S_{\text{A}}-S_{\text{B}}|}{\sqrt{\sigma_{\text{A}}^{2}+\sigma_{\text{B}}^{2}}}.$$

Here, $|S_{\text{A}}-S_{\text{B}}|$ is the difference in signal between two tissue regions A and B with different optical properties. The standard deviations of the two signals are denoted $\sigma_{\text{A}}$ and $\sigma_{\text{B}}$ . All subsequent use of the A and B subscripts, refers to the here mentioned two tissue regions. In the following subsections, CNR expressions for the detection methods chosen for this study are presented.

### 2.1 Shot-noise limited measurements and detector noise assumption

In the comparison presented below, of most interest is the best possible CNR which can be achieved by the different techniques. The detector noise for each of the techniques comes in slightly differently. Although it might be valuable to consider noise figures from commercially available cameras/detectors, detector and camera specifications are constantly improving. Further, we note that several of the methods can be operated in (or very close to) the shot-noise limit. For the spectral hole burning detection scheme, large area detectors (such as photomultiplier tubes) can have very low dark counts, allowing shot-noise limited measurements down to very low photon numbers. For the off-axis holography method, the setup can be configured such that the camera is operating in it’s shot noise limit [20]. For the photorefractive method there can be noise on the optical signal measured by the detector due to scattering of the reference beam in the photorefractive material as a result of defects and also beam fanning [35,36]. However, there are also photorefractive detection schemes which reduce the level of this noise [37,38]. In the best case scenario, the noise of the photorefractive signal measured on the detector will be dominated by the shot noise of the signal of interest. Finally, in the case of the speckle contrast method, the setup may, for certain cases, also be configured such that other sources of noise dominates over the detector noise. However, as will be shown later in Section 3, the speckle contrast method comparatively performs quite poorly, even when only considering noise from the light source. Adding detector noise would only reduce the achievable CNR further.

To summarize, the CNR values presented in Section 3. may overestimate the experimentally achievable CNR for both the photorefractive and speckle contrast methods due to sources of noise not included in the analysis.

### 2.2 Spectral hole burning filters

**Fig. 2.**
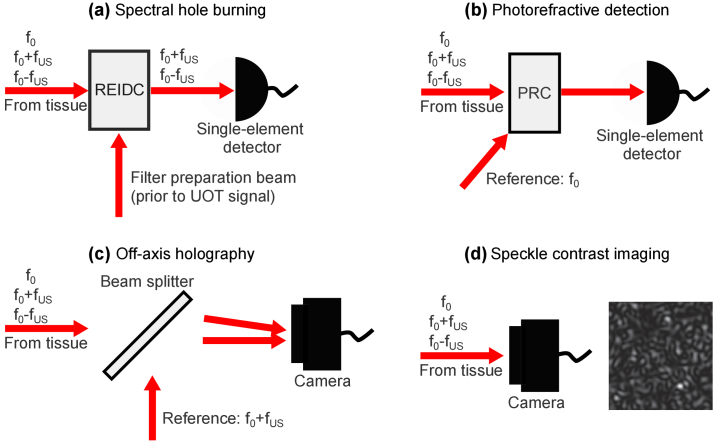
Simplified detection setups for the investigated methods. At the tissue output the photons are either untagged (at the original laser frequency $f_{0}$ ), or tagged (at frequency $f_{0}\pm f_{\text{US}}$ , where $f_{\text{US}}$ is the ultrasound frequency). **(a)** Spectral hole burning: Prior to the measurements, a seperate beam has created passband filters in a rare-earth-ion-doped crystal (REIDC). During the UOT measurements, the crystal transmits tagged photons and attenuates untagged photons. **(b)** Photorefractive detection: the interference between the reference and untagged photon field (at frequency $f_{0}$ ) generates an index grating in the photorefractive crystal (PRC). Part of the reference field is diffracted by the grating, and adapts the wave front of the untagged field. The two fields interfere on a single element detector. **(c)** Off-axis holography: the spatial interference between the reference and +1st order tagged fields, both at $f_{0}+f_{\text{US}}$ , is recorded. Subsequent Fourier transform, cropping, and inverse Fourier transform allows for reconstructing the tagged photon field. **(d)** Speckle contrast imaging: the speckle pattern is recorded with a single camera exposure. The decrease in speckle contrast caused by the ultrasound is calculated and used as the UOT signal.

The spectral hole burning method relies on high contrast spectral band-pass filters to remove the large background of untagged photons, allowing only tagged photons to reach the detector. The filters are created in cryogenically cooled rare-earth-ion-doped crystals using optical pumping techniques [11,39]. It is possible to create filters to detect tagged photons shifted towards both higher (+1st order) and lower (-1st order) frequencies [10], and such a setup is assumed in this paper. A simplified spectral hole burning setup is shown in Fig. 2(a).

The difference in the number of detected tagged photons from probing different tissue regions, gives the image contrast $=2S\eta_{\text{det}}|N_{T,\;A}-N_{T,\;B}|$ . Here, $N_{T,\;A}$ and $N_{T,\;B}$ , are the number of +1st order tagged photons emitted from the tissue per unit area for tissue region A and B, respectively. The tissue area from which photons are collected is denoted S , and it is assumed that the photon flux is even across S . The detection efficiency $\eta_{\text{det}}$ , which depends on the detector quantum efficiency and other setup specific parameters causing photon losses between the tissue and detection, is further described in Section 2.7.

Ideally, the filter would block all untagged photons. In reality, some untagged photons may be transmitted through the filter. The transmission of untagged photons through the filter is denoted $T_{\text{U}}$ . The spectral hole burning technique is ultimately limited by shot-noise. The noise (signal standard deviation) from probing a single tissue region, is then the square root of the total number of detected photons (tagged + untagged) from that region. By assuming that the number of untagged photons that are emitted from the tissue per unit area, $N_{\text{U}}$ , when probing tissue region A and B are equal, the shot-noise limited CNR can be written:

(2) $${\text{CNR}}_{\text{SHB}}=\sqrt{2S\eta_{\text{det}}}\frac{|N_{T,\;A}-N_{T,\;B}|}{\sqrt{N_{T,\;A}+N_{T,\;B}+T_{\text{U}}N_{\text{U}}}}.$$

By detecting photons from a larger tissue area, and by improving the detection efficiency and the filter attenuation, the CNR is improved. Depending on filter performance and imaging depth, either the tagged or untagged photons can dominate the noise, leading to different CNR scaling. The best reported attenuation of untagged photons is ∼30 dB ( $T_{\text{U}}∼10^{-3}$ ) using a 1.2 cm long ${\mathrm{Pr}}^{3+}$ : $Y_{2}$ ${\mathrm{SiO}}_{5}$ crystal [9,11]. Moreover, ${\mathrm{Tm}}^{3+}$ : ${\mathrm{LaF}}_{3}$ crystals are being investigated for the UOT application due to their ability to act as filters at the medically relevant wavelengths of 690 and 797 nm. For a 0.5% thulium (Tm) doping concentration, the 690 nm transition has an absorption coefficient of 15 ${\mathrm{cm}}^{-1}$ , and allows the burning of <1 MHz wide spectral holes [40]. For a ∼ 1.2 cm long crystal, the theoretical attenuation would be ∼80 dB. Thus, ${\mathrm{Tm}}^{3+}$ : ${\mathrm{LaF}}_{3}$ is a promising filter material candidate, but further material characterization is needed.

### 2.3 Photorefractive detection

The photorefractive method is based around materials exhibiting the photorefractive effect. The speckle field exiting the tissue and a reference beam are overlapped in the photorefractive material at an angle. The resulting interference generates an index grating. Part of the reference field is diffracted by this grating with efficiency $\eta_{\text{pr}}$ . The diffracted reference field replicates the wavefront of the speckle field, meaning that the two can now interfere on a large single-element detector. The setup can be configured to either detect tagged [24,26,29] or untagged [25,27,28] photons. Gross et al. state that theoretically the signal can be a factor of 2 higher when detecting tagged photons [29], which in principle allows for an increase in CNR by a factor of $\sqrt{2}$ . However, experimental implementations of both detection schemes (detecting either tagged or untagged photons) have found that the SNR was roughly equal [26,29]. We will hereafter assume that the untagged photons are detected. A simplified photorefractive setup is shown in Fig. 2(b).

By subtracting measurements with and without ultrasound modulation, the tagged photons can be indirectly detected, giving a signal $\Delta I≃2\eta_{\text{pr}}|E_{T}{|}^{2}$ [29] (prefactors have been omitted for convenience). The amplitude of the +1st order tagged photon field, $E_{T}$ , is assumed to be significantly smaller than the amplitude of the untagged field $E_{U}$ , and the grating efficiency low. The factor 2 appears because tagged photons shifted by both ± the ultrasound frequency are detected. In terms of tagged photons, the signal is $≃2S\eta_{\text{det}}\eta_{\text{pr}}N_{T}.$

The total intensity on the detector without ultrasound modulation is $I=|E_{U}+E_{D}{|}^{2}$ [29]. Here, $E_{D}$ is the amplitude of the diffracted field, and for low grating efficiencies, $E_{D}\approx \eta_{\text{pr}}E_{U}$ . Thus, re-writing the total intensity on the detector in terms of detected photons gives $S\eta_{\text{det}}N_{U}{\left(1+\eta_{\text{pr}}\right)}^{2}$ . The total number of detected photons is approximately equal when probing both with and without ultrasound, and for region A and B. The CNR is therefore written as:

(3) $${\text{CNR}}_{\text{PR}}=\sqrt{2S\eta_{\text{det}}}\frac{\eta_{\text{pr}}|N_{T,\;A}-N_{T,\;B}|}{\sqrt{N_{U}{\left(1+\eta_{\text{pr}}\right)}^{2}}}.$$

Thus, increasing the detection area and efficiency, and/or increasing $\eta_{\text{pr}}$ improves the CNR. For the photorefractive method to be useful for in vivo imaging, and for Eq. (3) to hold, the photorefractive response time has to be short compared with the speckle decorrelation time in tissue ( ∼ 0.1-1 ms [33]). Several experiments have been performed using materials with photorefractive response times much too long ( ∼100 ms) for in vivo measurements [25,27,28]. Materials with faster repose times have been investigated. Ramaz et al. [24] used a GaAs crystal that had a response time of 1 ms with $\eta_{\text{pr}}=15\%$ . Farahi et al. [41] used Te:SPS which operated in the red and near infrared with a few ms response time.

As discussed in Section 2.1, crystal imperfections (which induce scattering of the reference beam) and beam fanning [35,36] may be non-neglible noise sources, and are not included in Eq. (3).

### 2.4 Single-shot off-axis holography

In the single-shot off-axis holography method [20], the laser light is split into a reference and a signal beam. The signal beam is shifted by the ultrasound frequency using acousto-optic modulators, and directed onto the tissue. The output signal field (both tagged and untagged) and reference beam are overlapped on a camera, with the reference beam propagating at an angle relative to the signal beam. The interference between the signal field and reference field is recorded. The beating term (at the ultrasound frequency) between the untagged photon field and reference field averages out if the camera integration time is sufficiently long or an integer number of ultrasound periods. A two-dimensional (2D) fast Fourier transform (FFT) is then performed on the spatial interference pattern recorded. The terms related to the interference between the tagged photon and reference field are separated from the other terms in Fourier space. By cropping these regions which contain information on the tagged field and performing an inverse FFT, the tagged photon field can be retrieved. A simplified off-axis holography setup is shown in Fig. 2(c).

Liu et al. [20] presented an expression for the shot-noise limited SNR of their method when the untagged and reference field amplitudes are equal and much larger than the tagged field amplitude, and a rectangular iris is used before the camera. Based on their expression, we write the shot-noise limited CNR as:

(4) $${\text{CNR}}_{\text{HOL}}=\sqrt{N_{\text{px}}}\frac{\eta_{\text{det}}|{\bar{N}}_{T,\;A}-{\bar{N}}_{T,\;B}|/n}{\sqrt{4\eta_{\text{det}}({\bar{N}}_{T,\;A}+{\bar{N}}_{T,\;B})/n+2}}.$$

Here ${\bar{N}}_{T,\;A}$ and ${\bar{N}}_{T,\;B}$ are the average number of +1st order tagged photons per speckle at the tissue surface when probing tissue region A and B, respectively. The total number of camera pixels is denoted $N_{\text{px}}$ , and n is the number of camera pixels that cover each speckle, such that e.g. $\eta_{\text{det}}{\bar{N}}_{T,\;A}/n$ is the average number of detected tagged photons per pixel. The signal is maximized when the speckle size is 4 pixels wide [20]. Therefore, n=16 was assumed throughout this paper. The CNR can be improved by increasing the number of detector pixels which increases the number of detected speckles, and improving the detection efficiency. Depending on the average number of detected tagged photons per pixel, either the factor $4\eta_{\text{det}}({\bar{N}}_{T,\;A}+{\bar{N}}_{T,\;B})/n$ or 2 in the denominator of Eq. (4) determine the CNR scaling with imaging depth. The transition between the two scaling regimes occurs when the average tagged photon level per pixel is ∼1 . This can be conceptually understood from the fact that when the number tagged photons per pixel ≪1 , most pixels only contain noise, and does not contribute to the signal. Since all pixels still are used in the Fourier transform, they all contribute to the noise, while only a few of them contribute to the signal. When the number of tagged photons per pixel ≫1 all pixels contribute to both the signal and the noise. For single-shot off-axis holography the 2D FFT, cropping, and inverse 2D FFT has to be performed to obtain a single UOT signal. The method could thus get computationally heavy as the detector pixel count is increased.

### 2.5 Speckle contrast imaging

Speckle contrast imaging has been used extensively within biomedical optics, e.g. to measure blood flow [42]. The contrast of a speckle pattern, C , is defined as the standard deviation divided by the average value of the intensity. For UOT, the decrease in speckle contrast due to the interaction with an ultrasound field is used for imaging. The most common speckle method [14–16,19] uses a camera to record the speckle pattern, setting the exposure time to much longer than one ultrasound period (or an integer number of ultrasound periods). A simplified speckle contrast setup is shown in Fig. 2(d). If $N_{\text{T}}\ll N_{\text{U}}$ , and linearly polarized speckle patterns with close to unity contrast are considered (an ideal case), the contrast of the imaged speckle pattern can be written as [43]:

(5) $$C=1-\frac{2N_{\text{T}}}{N_{\text{U}}}.$$

Thus, the ratio of tagged to untagged photons determines the decreased speckle contrast which is the signal for this method. The difference in the ratio of tagged to untagged photons from probing tissue regions with different optical properties gives the image contrast. For our near unity contrast speckle patterns, only noise due to stochastic variation in speckle contrast will be considered. Following the algorithm in [44], speckle patterns where the speckle size was two pixels wide were simulated. The standard deviation of the contrast of many simulated speckle patterns was found to be $\approx \sqrt{1/N_{\text{px}}}$ . If the spread in the measured speckle contrasts follow a normal distribution, the CNR can be expressed as:

(6) $${\text{CNR}}_{\text{SC}}=\sqrt{2N_{\text{px}}}\frac{|N_{T,\;A}-N_{T,\;B}|}{N_{U}}.$$

By increasing the camera pixel count to sample more speckles, the noise is reduced, and the CNR improved. Note, Eq. (6) only includes noise due to stochastic variation in speckle contrast, and no other noise sources that will become relevant in CMOS and CCD detectors at low light levels and at high readout speeds. Also, ideal, polarized speckle patterns with close to unity contrast are considered. Lower speckle contrasts are generally measured experimentally [14,15,19]. Equation (6) should therefore rather be seen as an upper limit on the achievable CNR.

### 2.6 Simulating tagged and untagged photon levels

To evaluate the presented CNR equations, photon numbers were calculated from a hypothetical experiment in muscle tissue with an absorbing inclusion. While choosing a specific and highly medically relevant example can be valuable and may in the future be performed, it is the comparison between methods that is of most importance for this study. Therefore, our example was chosen to be a general imaging case, rather than attempting to estimate exact imaging depths for a specific medical application. The purpose of the example is to display the relative performance of the detection methods, and the scaling behavior of the CNR equations.

**Fig. 3.**
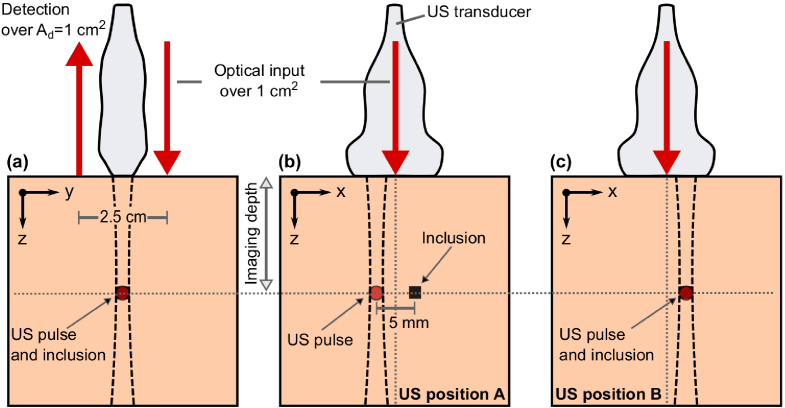
Schematic of simulation geometry. **(a)** y-z view **(b)** x-z view when the ultrasound (US) pulse is probing tissue region A, i.e., displaced 5 mm from the inclusion. **(c)** x-z view when the US pulse is probing tissue region B, i.e., overlapping with the inclusion. The CNR was calculated for different z-positions (imaging depths) of the ultrasound pulse and optical inclusion. Note, distances in the figure are not to scale.

For muscle tissue, the reduced scattering coefficient can be written as $\mu_{s}^{\prime }=a\;{\left(\lambda /500\text{nm}\right)}^{-b}$ [45], where b is the scattering power, and a is a tissue type dependent parameter. Two sets of literature values for (a,b) for muscle tissue (Alexandrakis et al [46] and Tromberg [45]) were averaged, which gave $\mu_{s}^{\prime }\approx 5$ ${\mathrm{cm}}^{-1}$ at λ=800 nm. An anisotropy factor of g=0.9 [45] was considered, which gave a scattering coefficient $\mu_{s}=50$ ${\mathrm{cm}}^{-1}$ . An absorption coefficient of $\mu_{a}=0.2$ ${\mathrm{cm}}^{-1}$ was used for the background medium. The inclusion had the same scattering properties as the background tissue, but the inclusion absorption was varied to consider absorption contrasts ( $\mu_{a,inclusion}/\mu_{a,background}$ ) between 1 and 2.5. The inclusion had dimensions of 3×3×3 ${\mathrm{mm}}^{3}$ , and was added at depth z (centered along y and displaced 2.5 mm from center along x ), see Fig. 3. It was assumed that 30×30 image voxels should be acquired in 1 s, with a 3×3×3 ${\mathrm{mm}}^{3}$ resolution (matching voxel and ultrasound volume), in a reflection geometry (Fig. 3). Although transmission geometries (optical input and detection on opposite sides of the sample) may be preferred for certain applications, e.g. breast imaging, we deem reflection geometries more generally useful.

To generate the tagged and untagged photon levels for the comparison, the geometry shown in Fig. 3 was simulated using a Monte Carlo (MC) and first principle photon tagging simulator [47,48], as described in Appendix A. The simulated signal levels have been validated against measurements on phantoms with tissue-like scattering (but low absorption) [49]. Using a 2 MPa peak pressure ultrasound pulse located either at position A, see Fig. 3(b), or position B see Fig. 3(c) the tagged and untagged light transmissions through the medium were simulated, thus a total of four transmissions. These transmissions were multiplied by the number of input photons ( $10^{15}$ ) to generate the number of tagged and untagged photons emitted through the detection area $A_{\text{d}}$ with the ultrasound at either position A or B. The average number of photons per speckle at the tissue surface was obtained by multiplying the number of photons emitted through the detection area $A_{d}$ by the speckle area. For the 800 nm wavelength, a 600 nm speckle diameter was used [50]. A simplification here is thus that the optical flux is even across the whole detection area $A_{\text{d}}$ . The z depth of the inclusion and ultrasound pulse (positions A and B) was varied to calculate the CNR as a function of imaging depth.

For our imaging example, the medical safety limit of 300 ${\mathrm{mW/cm}}^{2}$ at 800 nm (average radiation) [51] limits the maximum number of photons that can be used during the 1 s image acquisition to $∼10^{18}$ for a 1 ${\mathrm{cm}}^{2}$ input beam area. Thus, $10^{15}$ input photons can be allocated to each of the 900 measurement regions. The photons can be delivered with a single laser pulse, or using multiple lower energy pulses. For the spectral hole burning and photorefractive methods, 1D lines can be scanned using longer duration laser pulses to follow each ultrasound pulse as it propagates. For the spectral hole burning method, a pulsed scheme can also be used to acquire the image voxels one by one. For the spectral hole burning and photorefractive methods, it is possible to perform multiple averages for each voxel/line scan during the 1 s image acquisition. For the camera based methods the ultrasound pulse propagation is not resolvable due to the limited camera frame rate, and each image voxel is therefore acquired one by one. Performing multiple averages for each voxel is also limited by frame rate. Instead, for example, a ∼0.2 mJ pulse energy, ∼1 μ s pulse duration at 900 Hz could be used to deliver the $10^{15}$ input photons for each voxel. Alternatively, a long ultrasound pulses could be used to allow longer laser pulses with lower peak power. With conventional point-by-point B mode ultrasound pulses, this would come at the cost of decreased axial resolution. Recently however, structured ultrasound excitation schemes have been successfully demonstrated, which can recover mm axial resolution when using ultrasound pulses with long durations [23,30,31]. As the volume of these structured ultrasound pulses is larger than in voxel-by-voxel imaging, more photons are tagged; something all considered methods’ signal strength would benefit from. Nevertheless, in our imaging example, we consider a point-by-point ultrasound scheme.

### 2.7 Detection efficiency

The strong optical scattering by tissue means that photons will exit from a large tissue surface area and over all angles (we make the simplification that tissue is a Lambertian emitter [52], hence we assume photons emit at a projected solid angle of ∼π ). To increase the UOT signal, it is desirable to maximize the area and numerical aperture (NA) of the detection setup, and the detector quantum efficiency. The photons can be collected directly using lenses. Alternatively, light guides or fiber bundles in direct contact with the tissue, possibly followed by lenses, can be used, allowing separation between the detection setup and patient, and easier design of hand held probes. Light guides or fiber bundles have e.g. been used with the spectral hole burning [11], speckle contrast [19], photorefractive [28] methods. However, light guides partially suppress laser speckle contrast [53], which would have to be considered for speckle methods.

Identifying the optimal detection setup for each individual method is outside the scope of this paper. In the CNR calculations, it was simply assumed that the detection setups were limited by an NA of 0.7, corresponding to a $\sin^{-1}(0.7)=45^{\circ }$ collection angle. Thus, the signal loss due to the limited collection angle was set to $\sin^{2}\left(45^{\circ }\right)=0.5$ . The detection NA also affects the speckle size. As the resolution limit r of imaging a speckle on the tissue surface onto the camera scales as r=0.61λ/NA , and as photons exit the tissue with a large spread of angles, the smallest speckle area which can be recorded on the detection camera was assumed to be proportional to $1/{\text{NA}}^{2}$ . For the assumed NA, the speckle area thus increases by a factor 2. Although the limited NA decreases the intensity in a speckle, the number of photons per speckle at the tissue and at the detector are the same, because of the increased speckle area. This was considered for off-axis holography, where the number of photons per speckle is relevant.

The camera pixel count and the required number of speckles/pixel determines the tissue area from which camera based methods can collect photons from. To the best of the authors’ knowledge, current state of the art low noise, ∼ kHz frame rate cameras have a few Mega-pixel (Mpx) sensors. In the calculations, either a 1 Mpx or a 50 Mpx camera was used. The pixel quantum efficiency was assumed to be 60% . Typical off-axis holography setups use linearly polarized reference beams and half of the photons from the tissue contribute to the signal. For the speckle contrast method, $\eta_{\text{det}}$ is not present in the CNR equation [Eq. (6)], since it cancels out when taking the ratio between tagged and untagged photons. However, Eq. (6) is a simplified expression. At low light levels, noise sources not accounted for will affect the CNR, and it is important to optimize $\eta_{\text{det}}$ also for the speckle contrast method.

Crystals and detectors with 1 ${\mathrm{cm}}^{2}$ area are readily available, therefore the spectral hole burning and photorefractive methods were assumed to collect photons from a S=1 ${\mathrm{cm}}^{2}$ tissue area (which corresponds to $>10^{8}$ speckles). Photomultiplier tubes or multi-pixel photon counters are suitable detectors for the spectral hole burning method, and a detector quantum efficiency of 15% was used. The photorefractive method detects small changes on a relatively strong signal, and can e.g. use a photo diode with 80% detector quantum efficiency. Thus, taking the numerical aperture of collection optics into consideration, for the spectral hole burning, off-axis holography, and photorefractive methods, $\eta_{\text{det}}$ was assumed to be 7.5% , 30% and 40% , respectively.

## 3. Results and discussion

**Fig. 4.**
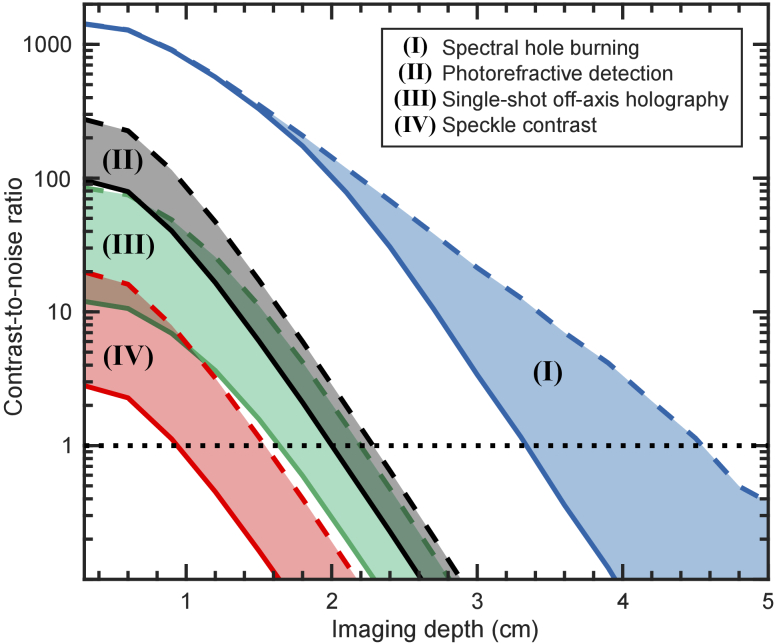
Contrast-to-noise ratios of the investigated methods for the imaging example presented in Section 2.6, for an inclusion absorption of $\mu_{a,inclusion}=0.4\;{cm}^{-1}$ , with $\mu_{a,background}=0.2\;{cm}^{-1}$ (an absorption contrast of 2). **(I)** Spectral hole burning assuming the transmission of untagged photons $T_{\text{U}}$ through the spectral filter is $T_{\text{U}}=10^{-3}$ (blue line) and $T_{\text{U}}=10^{-8}$ (blue dashed line). **(II)** Photorefractive detection assuming the photorefractive grating efficiency $\eta_{\text{pr}}$ is $\eta_{\text{pr}}=0.10$ (black line) and $\eta_{\text{pr}}=0.35$ (black dashed line). **(III)** Single-shot off-axis holography assuming the number of pixels $N_{\text{px}}$ in the detector camera is $N_{\text{px}}=1$ Mpx (green line) and $N_{\text{px}}=50$ Mpx (green dashed line). **(IV)** Speckle contrast assuming $N_{\text{px}}=1$ Mpx (red line) and $N_{\text{px}}=50$ Mpx (red dashed line). To be able to distinguish the absorption difference, the CNR has to be above 1, which is indicated by the horizontal black dotted line.

The CNR dependence on imaging depth for the four methods investigated is shown in Fig. 4. Since the tagged signal has traveled from the optical input to the ultrasound pulse and then to the detection output, the minimum geometrical path in centimeters for a given imaging depth z equals $2\sqrt{1.25^{2}+0.25^{2}+z^{2}}$ . This, for example, amounts to 3.9 cm at z=1.5 cm and 9.4 cm at z=4.5 cm. There are a wide range of simulation parameters that affect the imaging depths in Fig. 4. Changing the optical scattering and absorption coefficients, which enter exponentially, significantly modifies the predicted depths. There is a trade-off between ultrasound focus size (image resolution) and imaging depth. By increasing the probe beam area, the laser power could be increased while remaining within the medical safety limit. Smaller changes to e.g. the detection efficiency also weakly impact the CNR. However, the relative imaging performance of the methods and general change in CNR scaling with depth seen in Fig. 4 is expected to hold regardless of the parameter changes mentioned above.

For the particular measurement case considered here, the simulation predicts maximal imaging depths (where the CNR = 1) of 3.3 and 4.5 cm for the spectral hole burning method, with 30 dB ( $T_{\text{U}}=10^{-3}$ ) and 80 dB ( $T_{\text{U}}=10^{-8}$ ) filters, respectively. At depths <2 cm, the 30 and 80 dB filters give the same CNR, because the noise is dominated by tagged photons. At depths >2 cm, there is a change in scaling for the 30 dB filter, because the noise is dominated by untagged photons leaking through the filter. With 80 dB filters, the CNR remains in the favorable regime where the noise is dominated by the shot noise of the tagged photons down to ∼5 cm. The result shows the importance of identifying suitable filter materials with an attenuation of untagged photons beyond the 30 dB that have so far been used in experiments [9] in order to push the method beyond the current state of the art.

The photorefractive method reaches maximum imaging depths of 2.0−2.3 cm in the reflection geometry considered. The photorefractive method does not perform as well as the spectral hole burning method since the untagged photons dominate the noise for the investigated setup, see Eq. (3). The photorefractive method would benefit from a transmission geometry (since the ratio of tagged to untagged photons is often significantly higher). In the present case, for the 2.3 cm depth, the shortest path from input to ultrasound pulse to output is ∼5.3 cm. The conclusion drawn from the results is that the photorefractive setup could likely be used to image tissues significantly thicker than 5.3 cm in a transmission geometry (assuming all other simulation parameters are kept the same). It should again be noted that the imaging depths could be further extended by averaging, and increasing the ultrasound volume, etc. Lai et al. have demonstrated the great imaging capabilities of the photorefractive method in a transmission geometry with their measurements on a 9.4 cm thick phantom [28].

Single-shot off-axis holography reaches maximal imaging depths of 1.6 and 2.2 cm assuming 1 and 50 Mpx detectors, respectively. The average number of tagged photons detected per pixel is <1 regardless of imaging depth. Hence, the CNR scaling is always in the unfavorable regime (see Section 2.4). Off-axis holography measures fewer speckles compared with spectral hole burning in our example, and requires several camera pixels per speckle to resolve the interference fringes, yielding a CNR which is lower also for shallow imaging depths. Further, a camera with 1 Mpx resolution, 12-bit, recording at 1000 frames/s generates 1.5 GBytes of raw image data per second. This data has to be transferred from the camera. Holographic methods require performing 2D FFTs on matrices with $N_{\text{px}}$ elements to calculate each UOT signal. It can be challenging to avoid that camera based methods become slow compared with the spectral hole burning and photorefractive methods, which use single-point detectors and generate significantly less data, enabling a simpler data analysis. The time per UOT image frame is relevant, since the imaging depths in Fig. 4 can be further increased by averaging multiple frames. An advantage with camera based methods is that the detection is not limited to specific wavelengths. This gives more freedom for multi-color UOT targeting specific tissue molecules, which would offer a broader range of applications.

The speckle contrast method reaches maximum imaging depths of 0.9 and 1.5 cm for the 1 and 50 Mpx detectors, respectively. Already at comparatively shallow depths the ultrasound induced contrast reduction is not measurable due to the random properties of speckle patterns. Due to the optimistic assumptions when calculating the CNR for the speckle contrast method (see Section 2.5), and the assumption that camera noise does not factor in for this case, we expect the maximal imaging depths of the particular imaging case considered here to be even smaller. Thus, the method would perform poorly compared with the other investigated methods. Apart from the relative simple setup, the speckle method in Refs. [14,15,19] consequently does not seem optimal for UOT deep inside biological tissue.

**Fig. 5.**
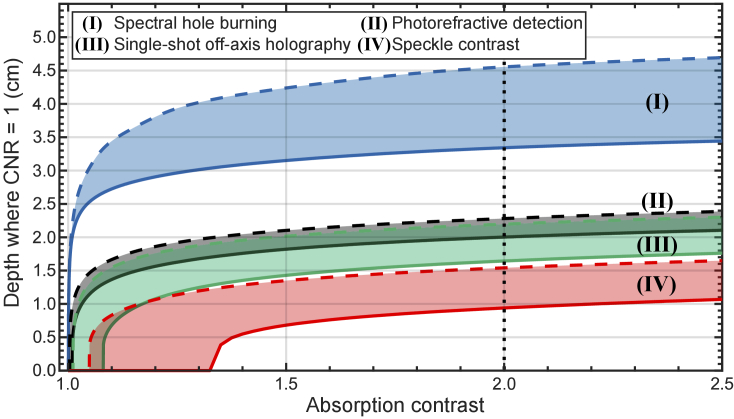
Depth at which the contrast-to-noise ratio is 1, as the inclusion absorption contrast ( $\mu_{a,inclusion}/\mu_{a,background}$ ) is varied from 1 to 2.5. The vertical dotted line indicates the absorption contrast used in Fig. 4. **(I)** Spectral hole burning assuming the transmission of untagged photons $T_{\text{U}}$ through the spectral filter is $T_{\text{U}}=10^{-3}$ (blue line) and $T_{\text{U}}=10^{-8}$ (blue dashed line). **(II)** Photorefractive detection assuming the photorefractive grating efficiency $\eta_{\text{pr}}$ is $\eta_{\text{pr}}=0.10$ (black line) and $\eta_{\text{pr}}=0.35$ (black dashed line). **(III)** Single-shot off-axis holography assuming the number of pixels $N_{\text{px}}$ in the detector camera is $N_{\text{px}}=1$ Mpx (green line) and $N_{\text{px}}=50$ Mpx (green dashed line). **(IV)** Speckle contrast assuming $N_{\text{px}}=1$ Mpx (red line) and $N_{\text{px}}=50$ Mpx (red dashed line).

So far, the discussion has been based on results with an absorption contrast of 2; in real applications, the absorption contrast between the inclusion and the background may be larger or smaller. Figure 5 shows the depth where the CNR reaches the critical value of 1, as a function of the absorption contrast, for the four methods considered here. Very small changes in absorption are measurable with the spectral hole burning and photorefractive methods. The same is true for the off axis holography method, if cameras with high pixel numbers are used. The speckle contrast method is more limited in terms of what absorption contrast is detectable. Note that for all methods the depth at which CNR = 1 increases by approximately the same amount (4-5 mm) when the absorption contrasts increases from 1.5 to 2.5.

We have devoted extensive efforts into verifying the number of tagged and untagged photons from our simulations experimentally [11,47] and work is ongoing to experimentally validate the CNR for tissue phantoms with inclusions. In principle, comparative experiments done by groups which specialize in each technique could be done using standardized phantoms. Such work could more firmly validate the results presented here.

In addition to CNR, many other aspects of the different methods may be relevant. Table 1 is an effort to list a number of relevant aspects for the four techniques. A few comments are given below.

**Table 1.** Broader comparison of the different techniques.

	Spectral hole burning	Photorefractive detection	Single-shot off axis holography	Speckle contrast
**Transmission/reflection imaging geometries?**	Favors transmission (transmission alleviates requirement for high contrast filter)	Favors transmission	Independent	Favors transmission
**Limiting expense**	Cryostat and frequency stabilized light source (high cost)	Photorefractive material and vibration isolation (medium cost)	High pixel count and high frame rate camera (medium cost)	High pixel count and high frame rate camera (medium cost)
**Technological advances** **which will make technique** **realisable**	Crystal filters and cryostats	Photorefractive material	Camera technology	Camera technology
**Laser frequency stability requirement**	High	Low	Low	Low
**Optical wavelength flexibility**	Limited to where suitable rare-earth ion-doped crystals exist	Limited to where materials exhibit photorefractive effect (Te:SPS has a broad wavelength range)	Not limited to specific wavelength	Not limited to specific wavelength
**Sensitivity to speckle decorrelation**	Insensitive	Sensitive	Sensitive	Sensitive
**Data generated per frame**	Low	Low	High (high bandwidth data stream required)	High (high bandwidth data stream required)
**Etendue Increases with:**	Larger crystal size	Larger photorefractive material size	Increasing number of pixels (also increases data generated)	Increasing number of pixels (also Increases data generated)

Firstly, for the reflection geometry used in our calculations, the untagged photon level is almost independent of imaging depth. The number of tagged photons depends strongly on both geometrical factors and imaging depth due to the exponential attenuation by tissue. For a transmission geometry, both the tagged and untagged photon levels decrease with the thickness of the imaged tissue. Hence, a transmission geometry (if compatible with the medical application) would be advantageous for the photorefractive method, the speckle contrast method, or for a spectral hole burning setup with limited filter attenuation. Further theoretical studies are required to estimate the performance of the different method in a transmission geometry.

Both of the camera techniques require cameras with high pixel count and high frame rates. In recent years, the performance and availability of such cameras has been improving. In the case of spectral hole burning setups, a cryostat and a frequency stabilized laser are required, which are more expensive and significantly less portable when compared to the components required by the other methods. Hence, any UOT medical device implemented using spectral hole burning as the imaging filter method will be more expensive and take more hospital space than other methods.

Finally, there are methods not covered in this paper that could potentially perform well. For example, the recently developed laser feedback method for UOT has shown interesting experimental results [32].

## 4. Conclusion

We have presented a theoretical study comparing the contrast-to-noise ratio (CNR) of the spectral hole burning, single-shot off-axis holography, speckle contrast, and photorefractive UOT methods, with the goal of better understanding the potential imaging performance of the many methods presented in the literature. Our results show that, out of the techniques compared, the spectral hole burning method has the potential to reach the largest imaging depths, since the method can have better CNR scaling compared with the other methods, and also has excellent etendue. However, the spectral hole burning technique is restricted to using wavelengths where it is possible to create high contrast filters. While there is a large number of crystal host-dopant combinations that absorb at medically relevant wavelengths, only a handful with suitable properties have been identified so far. Furthermore, efforts have to be directed towards the development of high contrast filters at these wavelengths, and shrinking the bulky cryogenic systems required to cool the filter crystals.

The single-shot off-axis holography method has an unfavorable change in CNR scaling when the average tagged photon count per pixel drops below ∼1 , which occur already at shallow tissue depths. Camera based methods are in general slow and currently suffer from lower etendue (due to limited camera pixel count at ∼ kHz readout rates), but has more flexibility regarding optical wavelength selection. The photorefractive method has large etendue, but, for the setup investigated in this paper, the noise is dominated by untagged photons, which has a negative effect on the achievable imaging depth. Moreover, photorefractive materials with faster response times should ideally be identified. We find that the frequently used speckle contrast method has poor CNR compared with the other investigated methods, and may therefore be less useful for imaging at large depths.

While higher CNR is preferable in all applications, there are many additional factors, see Table 1, which may ultimately determine how viable UOT devices are as imaging devices for medical diagnosis. There may be specific medical use cases where, for example, a cheaper system with shallower imaging depths has adequate imaging capabilities for diagnosis. Further side by side development of various methods may therefore be desirable.

## Figures and Tables

**Fig. 6.**
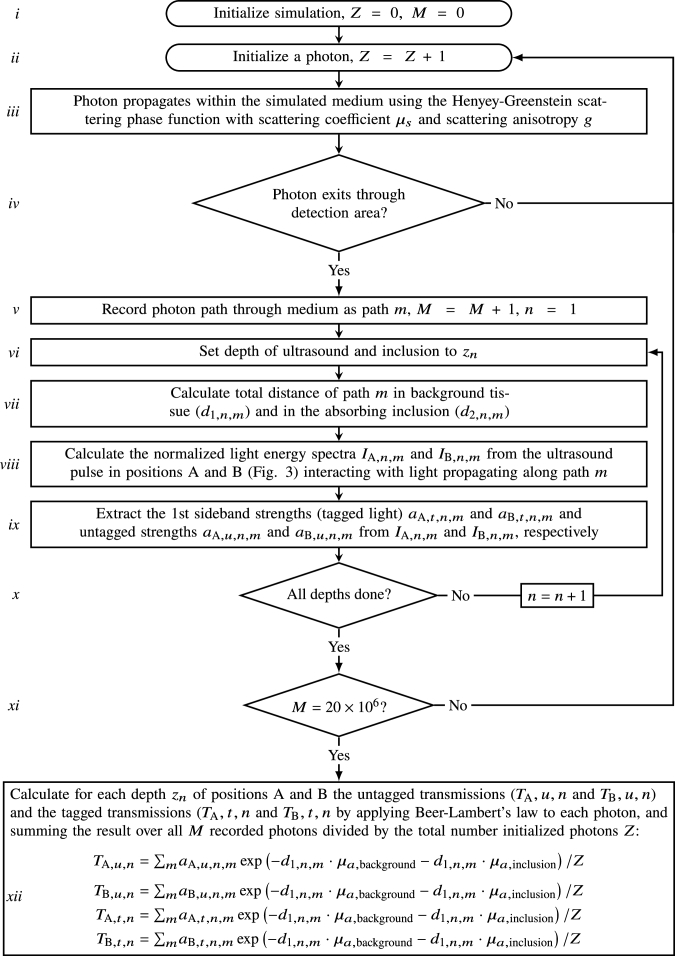
Overview of the simulation steps in the Monte-Carlo and photon tagging simulations. Symmetries were used in the actual code to speed up the simulations.

## Data Availability

No experimental data were generated or analyzed in the presented research. Computer code and a script for calculating and plotting the results contained in Fig. 4 are available in Ref. [54].

## Appendix: architecture of the Monte-Carlo simulations

The Monte Carlo (MC) and photon tagging simulator described in Refs. [47,48] was used to calculate the tagged and untagged photon levels at the tissue output. These were then used to calculate the CNR for all methods (the simulation code is provided online at Ref. [54]). The MC simulation domain was a 10×10×10 ${\mathrm{cm}}^{3}$ cuboid with $\mu_{s}=50$ ${\mathrm{cm}}^{-1}$ , g=0.9 , and refractive index of 1.3. In total, $Z=5.2\times 10^{9}$ individual photon packets were launched from a top hat distributed source with a circular area of 1 ${\mathrm{cm}}^{2}$ . A total of $M=20\times 10^{6}$ photon packets were detected through a circular area, $A_{\text{d}}=1$ ${\mathrm{cm}}^{2}$ , with its center 2.5 cm from the input source center, and these photons’ paths were stored. An illustration of the main calculation flow is shown in Fig. 6. The interaction between the ultrasound and a photon is only calculated if the photon reaches the detector (for clarity, not all photons which reach the detector are affected by the ultrasound). For all calculations, an ultrasound pressure field following a Gaussian distribution with dimensions 3×3×3 ${\mathrm{mm}}^{3}$ (full width at half maximum), 2 MPa peak pressure, and 3 MHz frequency was used. The modulated and normalized light spectra $I_{A,n,m}$ and $I_{B,n,m}$ was then calculated from each path m with the ultrasound at either position A or B (see Fig. 3) at depth $z_{n}$ . From these spectra, the untagged photon strengths $a_{A,u,k,j}$ and $a_{B,u,k,j}$ and the first sideband strengths (the tagged light) $a_{A,t,k,j}$ and $a_{B,t,k,j}$ , are calculated by numerical integration of the spectra over either the center or sideband frequency. As each photon path m has a random phase $\omega_{m}$ in relation to the others, the contribution from each path may be added without regard to interference since $|\sum_{m}^{M}A_{m}\exp(i\omega_{m}){|}^{2}\approx \sum_{m}^{M}|A_{m}{|}^{2}$ if $\omega_{m}$ is uniformly random on the interval [0,2π] and M is large [55]. This allows for the piece wise calculation of the transmission, instead of requiring that the total electric field at the detector is calculated before calculating the intensity. For each position A and B at depth $z_{n}$ , the photon path length in the background region $d_{1,n,m}$ and in the inclusion $d_{2,n,m}$ is calculated. The effect of absorption was then added for each photon path using Beer-Lambert’s law, where after the total transmissions $T_{A,u,n}$ , $T_{B,u,n}$ , $T_{A,t,n}$ and $T_{B,t,n}$ , were calculated by summing the individual transmissions of all M paths and dividing by the total number of initialized photons, see Fig. 6-(*xii*).
